# Supplementary material for: Reconciling Mining with the Conservation of Cave Biodiversity: A Quantitative Baseline to Help Establish Conservation Priorities
Source: PLoS One. 2016 Dec 20;11(12):e0168348. doi: 10.1371/journal.pone.0168348 (PMC5173368; doi:10.1371/journal.pone.0168348)
Supplement: S1 Dataset — (ZIP) [file pone.0168348.s002.zip › Taxa/Serra Norte/SN_2013/N4E/Lista N4E-88.pdf]

CAVIDADE N4E-0088

Classe	Ordem	Fam/Outros	Gên/Outros	Espécie	S-U	U-S	Zon.
Annelida	Oligochaeta			jovem		2	E
Arachnida	Acari	(Parasitengonina)		sp.14		X	E
Arachnida	Acari	(Parasitengonina)		sp.15		X	E
Arachnida	Acari	Veigaiidae		sp.1		X	E
Arachnida	Amblypygi	Charinidae	*Charinus*	sp.1		5	P
Arachnida	Amblypygi	Phrynidae	*Heterophrynus*	*longicornis*	3		E/P
Arachnida	Araneae	Ochyroceratidae	*Speocera*	sp.1		X	E
Arachnida	Araneae	Pholcidae	*Mesabolivar*	*aurantiacus*	X	X	E
Arachnida	Araneae	Pholcidae	*Mesabolivar*	sp.1	X		E
Arachnida	Araneae	Pisauridae		jovem	4		P
Arachnida	Araneae	Theridiidae	*Theridion*	sp.4	X	X	E
Arachnida	Araneae	Theridiosomatidae	*Plato*	sp.1	X	X	E/P
Arachnida	Opiliones	Sclerosomatidae	*Prionostema*	sp.1	4		E
Arachnida	Opiliones	Stygnidae	*Protimesius*	*laevis*	1		E
Arachnida	Opiliones	Stygnidae	*Protimesius*	*gracilis*	1		E
Diplopoda	Polydesmida	Cyrtodesmidae		sp.1	X		E
Entognatha	Collembola	Paronellidae		sp.1		X	E
Insecta	Coleoptera	Staphylinidae	Pselaphinae	sp.2		X	E
Insecta	Diptera	Tipulidae	Tipulinae	sp.	X	X	E/P
Insecta	Heteroptera	Veliidae	*Rhagovelia*	sp.		X	P
Insecta	Homoptera	Cixiidae		jovem	X		E
Insecta	Hymenoptera	Formicidae	*Brachymyrmex*	sp.1	X		E
Insecta	Hymenoptera	Formicidae	*Camponotus*	sp.1		X	E
Insecta	Hymenoptera	Formicidae	*Hypoponera*	sp.1		X	E
Insecta	Hymenoptera	Formicidae	*Pachycondyla*	sp.2		X	E
Insecta	Hymenoptera	Formicidae	*Paratrechina*	sp.1		X	P
Insecta	Hymenoptera	Formicidae	*Paratrechina*	sp.2		X	E
Insecta	Hymenoptera	Formicidae	*Solenopsis*	sp.1		X	E
Insecta	Orthoptera	Phalangopsidae	*Paraclodes*	sp.	3	1	E
Insecta	Orthoptera	Phalangopsidae	*Phalangopsis*	sp.	4	10	P
Insecta	Psocoptera			jovem		X	E
Gastropoda	Pulmonata	Systrophiidae	*Happia*	sp.	X		E
Malacostraca	Decapoda	Pseudothelphusidae		sp.1	1		
Symphyla		Scutigerellidae		sp.	X	X	E
Amphibia	Anura	Strabomantidae	*Pristimantis*	cf. *fenestratus*	14		E/P
Mammalia	Chiroptera	Furipteridae	*Furipterus*	*horrens*		30	E/P
